# Efficiency of energy funneling in the photosystem II supercomplex of higher plants†

**DOI:** 10.1039/c5sc04296h

**Published:** 2016-02-29

**Authors:** Christoph Kreisbeck, Alán Aspuru-Guzik

**Affiliations:** a Department of Chemistry and Chemical Biology, Harvard University Cambridge MA USA aspuru@chemistry.harvard.edu christophkreisbeck@gmail.com

## Abstract

The investigation of energy transfer properties in photosynthetic multi-protein networks gives insight into their underlying design principles. Here, we discuss the excitonic energy transfer mechanisms of the photosystem II (PS-II) C_2_S_2_M_2_ supercomplex, which is the largest isolated functional unit of the photosynthetic apparatus of higher plants. Despite the lack of a definite energy gradient in C_2_S_2_M_2_, we show that the energy transfer is directed by relaxation to low energy states. C_2_S_2_M_2_ is not organized to form pathways with strict energetically downhill transfer, which has direct consequences for the transfer efficiency, transfer pathways and transfer limiting steps. The exciton dynamics is sensitive to small changes in the energetic layout which, for instance, are induced by the reorganization of vibrational coordinates. In order to incorporate the reorganization process in our numerical simulations, we go beyond rate equations and use the hierarchically coupled equation of motion approach (HEOM). While transfer from the peripheral antenna to the proteins in proximity to the reaction center occurs on a faster time scale, the final step of the energy transfer to the RC core is rather slow, and thus the limiting step in the transfer chain. Our findings suggest that the structure of the PS-II supercomplex guarantees photoprotection rather than optimized efficiency.

## Abstract 2

Energy transfer in the C_2_S_2_M_2_ supercomplex is sensitive to structural modulations induced *e.g.* by the reorganization process, and is limited by the uphill transfer from CP47 to the RC core.

## Graphical abstract

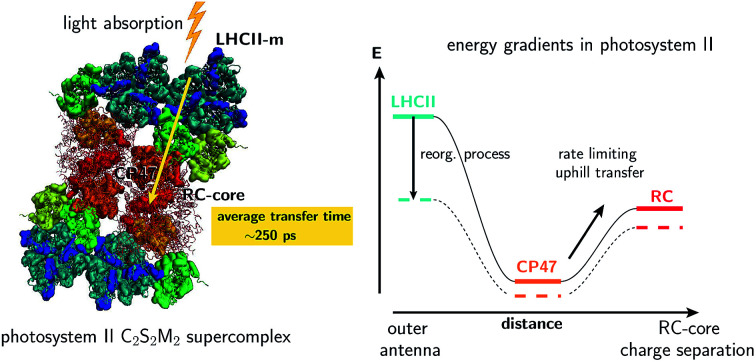

## 1 Introduction

Photosynthesis, in which light is absorbed and converted into chemical energy, is the most important process in nature. In higher plants the light-harvesting machinery is assembled of C_2_S_2_M_2_ supercomplexes and networks of LHCII pigment-proteins^1–4^ located in the grana membrane. The C_2_S_2_M_2_ supercomplex is formed by a dimeric photosystem II (PS-II) with loosely bound LHCII trimers and several minor complexes.^5^ Energy transfer to the reaction center (RC) core pigments of PS-II, in which the primary step of charge separation initializes an avalanche of photochemical reactions,^6–8^ reaches remarkable efficiencies of up to 90%.^9^ However, it remains unclear of how such high efficiencies can be achieved in large and disordered systems. In contrast to the photosynthetic apparatus of green sulfur bacteria, in which fast transfer is guaranteed by efficient energy funneling,^10^ microscopically derived Hamiltonians do not predict a definite energy gradient among the individual proteins of the C_2_S_2_M_2_ supercomplex.^11–15^

Previous works describe the transfer kinetics with phenomenological models, and extract certain decay components, such as the migration time (average time that it takes for an excitation to reach the RC) and trapping time, by fitting to fluorescence decay lines.^16–19^ Several rate limiting models are discussed in the literature.^17,18,20,21^ Recent studies favor the so-called transfer-to-trap limited kinetic model^12,16–18^ in which the transfer rate from the antenna complexes of PS-II to the RC is proposed to be the transfer-limiting step. However, different kinetic models can be fitted equally well to measured fluorescence decay curves,^22^ and structure-based models of energy transfer become necessary to shed light on the underlying transfer mechanisms. The first microscopic simulations of the exciton dynamics in the C_2_S_2_M_2_ supercomplex show that the overall transfer is driven by a complex interplay of multiple rates rather than through a single transfer-limiting step.^22^

In pigment–protein complexes, the directionality of energy transfer is driven by energy relaxation. Variations in the energy bands of the individual proteins in C_2_S_2_M_2_ are not as distinct as in other photosynthetic systems. Nevertheless, the energy gradient in C_2_S_2_M_2_ is not completely flat, and the pigments form a certain structure in the energetic layout. For example, CP43 and CP47 are lower in energy than the LHCII antenna complexes.^11,12,14^ However, energy transfer in C_2_S_2_M_2_ is not a cascade of downhill steps toward the reaction center. Actually, the pigments in the proximity of the RC core are the energetically lowest ones.^23^ Therefore, the last transfer step to the trap needs to overcome an energy barrier which supports the proposed transfer-to-trap limited exciton dynamics in C_2_S_2_M_2_. The transfer-limiting step to the RC core pigments, which is not anticipated in previous structure-based simulations,^22^ becomes more evident once we include the recently derived Hamiltonian of CP29.^15^ The latter is substituted in ref. 22 by an LHCII monomer. We show that the minor complex CP29 modifies the pathway of energy flow and yields a relaxation channel which drives energy from the peripheral antenna towards pigments closer to PS-II.

The transfer properties are sensitive to small structural modulations, which is an immediate consequence induced by the flat energy gradient. There are several mechanisms that induce changes in the energetic structure. These include static disorder, in which site energies are subjected to random fluctuations on much slower time scales than the exciton dynamics, or the reorganization process, in which vibrational coordinates relax to a new equilibrium position after a vertical Franck–Condon transition to the excited state energy potential surface.^24^ During this process, the reorganization energy is dissipated in the protein environment. While the transfer times of an ensemble of individual disorder realizations are randomly distributed around some average value,^22^ the reorganization process is a systematic effect pertaining to the dynamics in all realizations in the same way. Also the ratio of chlorophyll a *versus* chlorophyll b affects energy gradients. For example, *Chlamydomonas*-LHCII, found in species of green algae, does have a reduced chlorophyll a/b ratio when compared to the LHCII of higher plants, implying a blue shift in the absorption spectra.^25^

Due to the lack of the computational capability to carry out accurate calculations of the exciton dynamics, previous simulations of transfer time-scales in light-harvesting complexes (LHCs) employ a combined modified Redfield/generalized Förster rate equation approach.^22,26–29^ However, the combined modified Redfield/generalized Förster lacks the ability to simulate the reorganization process. In addition, those models provide an *ad hoc* description of dynamic localization, and depend on an empirical cut-off parameter. Recently, a non-Markovian (ZOFE) quantum master equation description has been employed to investigate the robustness of transfer efficiency and the importance of vibrationally enhanced transfer in PS-II.^30^ Here, we perform accurate simulations based on the hierarchically coupled equations of motion approach (HEOM),^31–35^ which accurately incorporates the reorganization process and works for a wide parameter range for the coupling strength to the environment.

Since the computational complexity of HEOM scales exponentially with increasing system size, novel algorithms based on optimized parallelization schemes have been developed.^36–38^ The most efficient implementation^36^ employs the high computational throughput provided by modern graphics processing units (GPUs), for which a cloud computing version is hosted on http://nanohub.org.^39^ GPU-HEOM is bound to the available GPU memory, and simulations are limited to intermediate-sized systems. Here, we overcome the memory limitation by using *QMaster*,^38^ which runs on various hardware architectures including GPUs and high memory multi-core CPU architectures. We make use of the large CPU memory to benchmark the convergence of the hierarchy depth and use the high computational throughput of the GPUs for production runs.

In Section 2 we outline the structure of the Frenkel exciton model for energy transfer in C_2_S_2_M_2_. The technical aspects of the HEOM approach are stated in Section 3. After that, we continue with the discussion of the time-scales of inter-protein transfer in the PS-II supercomplex (see Section 4). Finally, we investigate the impact of variations in the energetic structure of C_2_S_2_M_2_ on the transfer pathways and the transfer efficiency.

## 2 Exciton model

**Fig. 1:**
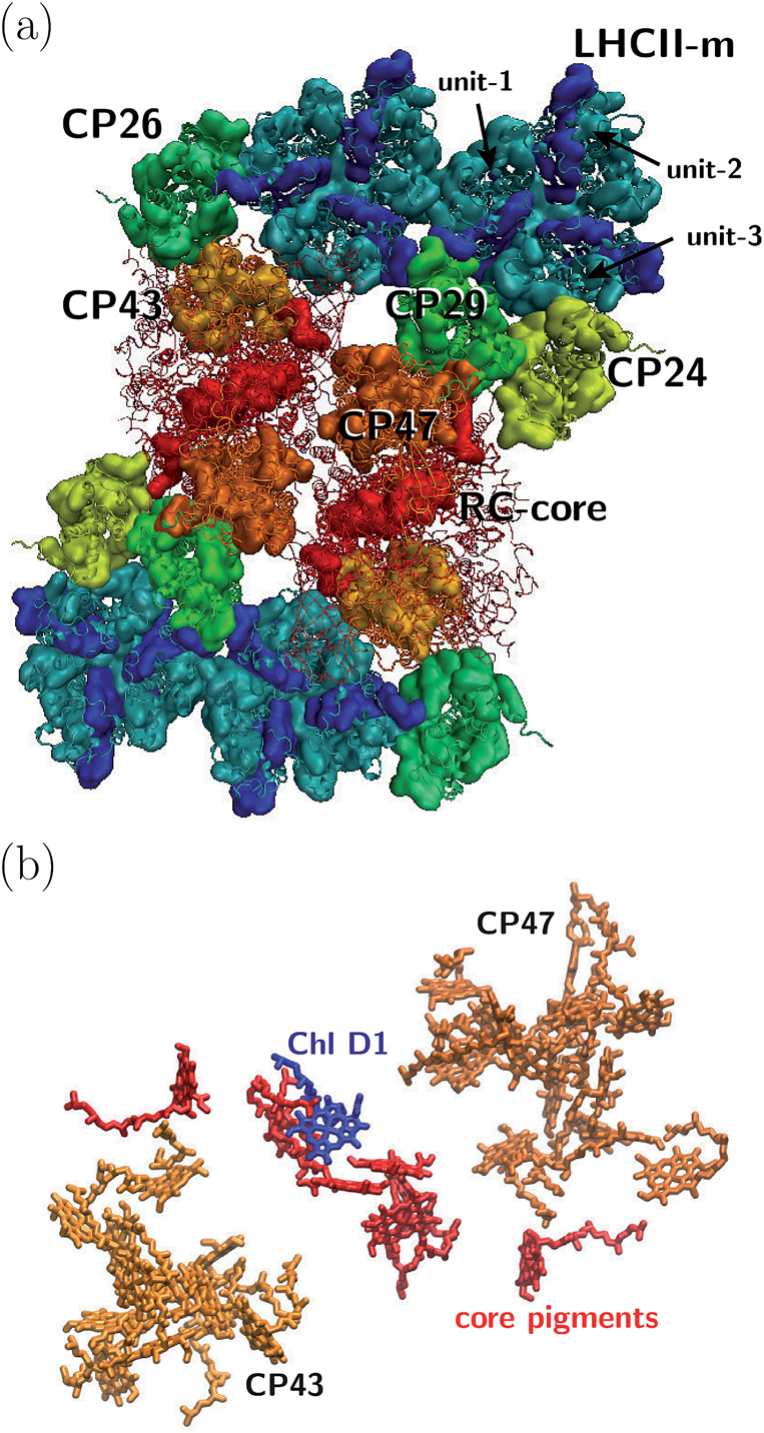
(a) Sketch of the protein structure of the C_2_S_2_M_2_ supercomplex. The multiprotein complex contains 4 LHCII trimers and the minor complexes CP24, CP26 and CP29 which are connected to the PS-II.^5^ (b) Assembly of the pigments of PS-II, composed of CP43, CP47 and the RC core. The primary step of charge separation is initiated through excitation of pigment Chl_D1_ (see eqn (1)).

The orientation of the individual proteins of the C_2_S_2_M_2_ supercomplex is determined by a projection map at 12 Å resolution.^5^ The C_2_S_2_M_2_ supercomplex, the structure of which is depicted in Fig. 1a, comprises four LHCII trimers, six minor light-harvesting complexes and a dimeric PS-II core complex. Absorbed light in the outer LHCII antenna complexes is transferred *via* the minor complexes CP24, CP26, and CP29 to CP47 and CP43 of PS-II. The transfer process is completed by irreversible charge separation triggered in the RC core. Electron transfer in the RC core is described phenomenologically by radical pair states RP1, RP2 and RP3.^12,40^ We assume that the primary step of charge separation is initiated through the electronically excited core pigment Chl_D1_ (the location of Chl_D1_ in PS-II is illustrated in Fig. 1b) and described by the rate equation1
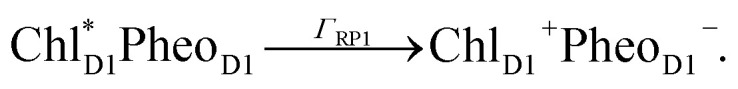


We neglect backward rates since fluorescence decay lines suggest that charge recombination occurs on a much slower time scale than primary electron transfer.^12^ Within this limit we model primary charge separation as irreversible exciton trapping. In the literature, more sophisticated models are also discussed, which include multiple pathways of charge separation.^41,42^

We describe energy transfer in the C_2_S_2_M_2_ supercomplex within a Frenkel exciton Hamiltonian, for which we assume that only one of the pigments is excited at once. The Hamiltonian of the single exciton manifold reads2
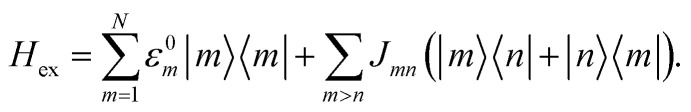


Here |*m*〉 denotes the state in which pigment *m* is excited while the other pigments remain in the electronic ground state. For the inter-site couplings *J*_*mn*_ we distinguish between intra-complex and inter-complex coupling terms, depending on whether or not pigments *m* and *n* are located within the same protein. We use the same parameters for the exciton Hamiltonian as in a previous study by Bennett *et al.* in ref. 22, in which the Hamiltonian for C_2_S_2_M_2_ is constructed as follows: the exciton system for the individual proteins of the LHCII-trimer, CP43, CP47, and the RC-core are taken from the literature,^11–14,43^ while the inter-protein couplings are calculated using a dipole–dipole approximation. Since the structures of the minor complexes CP24 and CP26 have not yet been resolved, they are substituted by LHCII monomers. Although the exciton dynamics within the minor complexes are shown to be roughly the same as the dynamics within LHCII,^11,44^ we remark that this replacement may affect the transfer pathways. In ref. 22, CP29 is also replaced by an LHCII monomer (without pigment Chl 605). However, recently the Hamiltonian for CP29 has been resolved.^15^ In order to isolate how much the new CP29 Hamiltonian influences transfer and to compare the HEOM results with previous approximate modified Redfield/generalized Förster simulations,^22^ we carry out calculations for both models: (i) with the CP29 Hamiltonian and (ii) with the LHCII monomer substitution.

The pigments are coupled to the protein environment modeled by a set of independent harmonic oscillators3
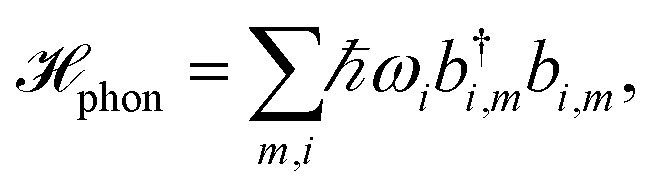
and we assume a linear coupling of the exciton system to the vibrations4
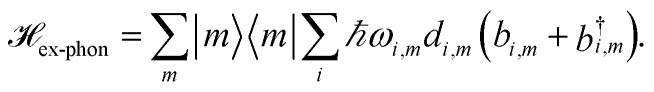


The reorganization energy 
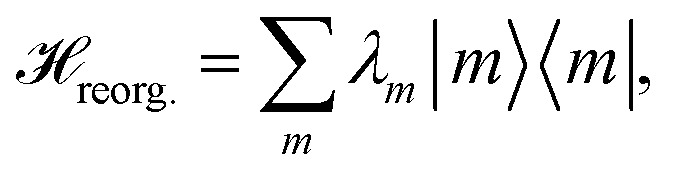
 with 
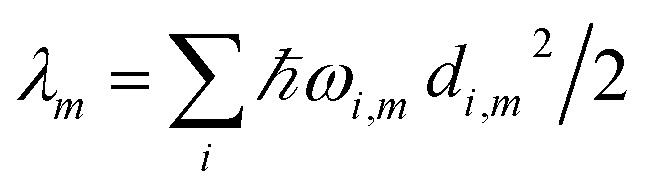
 is added to the exciton Hamiltonian eqn (2). We define the site energies as *ε*_*m*_ = *ε*^0^_*m*_ + *λ*_*m*_. The phonon mode-dependent coupling strength is captured by the spectral density5
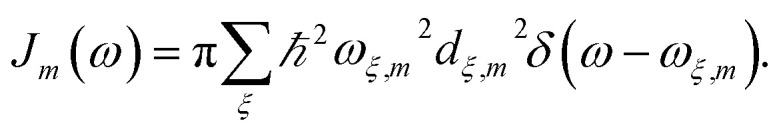


Frequently, the reorganization energy and the spectral density are assumed to be site independent. However, for the C_2_S_2_M_2_ supercomplex each individual protein has its own reorganization energies and own form for the spectral density.^22^ The spectral density for LHCII is extracted from fluorescence line narrowing spectra. Since the experimental spectra cannot distinguish between the Chla and Chlb pigments, we assume the same spectral density, composed of 48 vibrational peaks, for both.^45,46^ Transfer times are not much affected by the structures in the spectral density, and a coarse-grained Drude–Lorentz spectral density is appropriate to describe energy transfer in LHCII.^38^ More details about how the parameters for the coarse-grained spectral density are obtained can be found in ref. 38. Microscopic details for the spectral densities of the minor complexes and PS-II are not known. The structure of CP29 is similar to that of an LHCII monomer. Thus, we assume that the spectral density of CP29 can be substituted with the LHCII spectral density.^15,22^ For CP47 and the RC core pigments, *λ* = 38.64 cm^−1^ and *λ* = 50.23 cm^−1^ are respectively suggested as reasonable values for the reorganization energy.^12^ For the RC core pigments a higher reorganization energy is also discussed.^41,47^ The explicit form and parameters for the spectral densities used in this manuscript are given in the ESI.†

We include the primary step of charge separation phenomenologically as irreversible population trapping, which we incorporate by anti-Hermitian parts in the Hamiltonian

6 ℋ_trap_ = −i*ħΓ*_RP1_/2|Chl_D1_〉〈Chl_D1_|,

where *Γ*_RP1_ defines the rate of the primary charge separation. In a similar way we incorporate exciton losses7
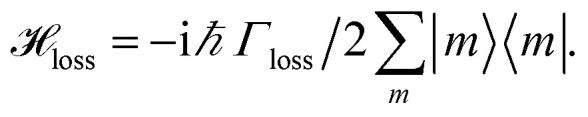
where we assume exciton lifetimes of (*Γ*_loss_)^−1^ = 2 ns. We characterize transfer properties by the transfer efficiency8
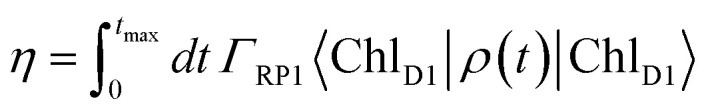
and average transfer time9
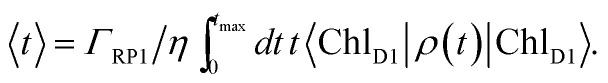


For numerical evaluations, we replace the upper integration limit by *t*_max_, which is chosen such that the total population within the pigments of the C_2_S_2_M_2_ supercomplex has dropped below 0.001.

## 3 Method

We evaluate the exciton dynamics within the hierarchically coupled equation of motion (HEOM) method.^31,34,35^ HEOM is an open quantum system approach which treats the coupling to the vibrational modes as a bath. The time evolution of the total density operator *R*(*t*), which characterizes the degrees of freedom of the exciton system as well as the ones of the phonon bath, is governed by the Liouville equation10
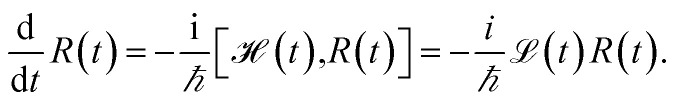


We assume that the total density operator *R*(*t*) factorizes at initial time *t*_0_ = 0 in excitonic and vibrational degrees of freedom *R*(*t*_0_) = *ρ*(*t*_0_) ⊗ *ρ*_phon_(*t*_0_). We trace out the vibrational degrees of freedom, and get the time evolution for the reduced density matrix *ρ*(*t*) describing the exciton degrees of freedom only:11
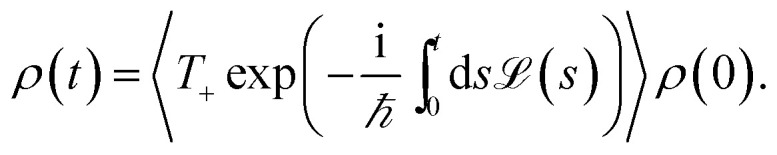


By employing a second order cumulant expansion, using a Drude–Lorentz spectral density 
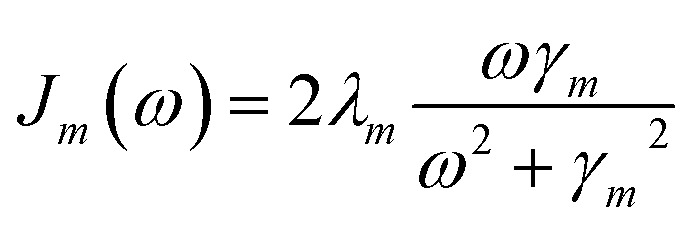
 in combination with a high temperature approximation *ħγ*_*m*_/*k*_B_*T* < 1, we cast the time non-local eqn (11) into a hierarchy of coupled time local equations of motion:12
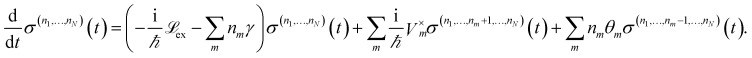
where we define *ρ*(*t*) = *σ*^(0,…,0)^(*t*), *V*^×^_*m*_• = [*V*_*m*_,•], *V*^o^_*m*_• = {*V*_*m*_,•}, 
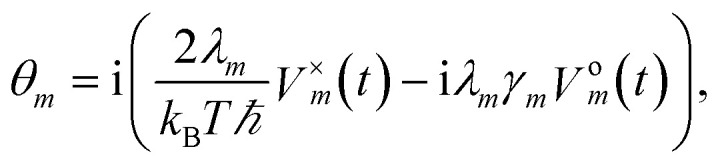
 and *V*_*m*_ = |*m*〉〈*m*|. The hierarchy can be truncated for a sufficiently large hierarchy level 
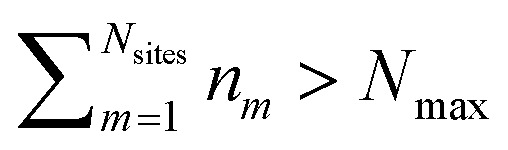
. Convergence of the hierarchy can be tested by comparing deviations in the dynamics with increasing truncation level.

To increase the accuracy of the high temperature approximation (HTA) of HEOM,^48^ we include additional correction terms^49^ for which we replace13
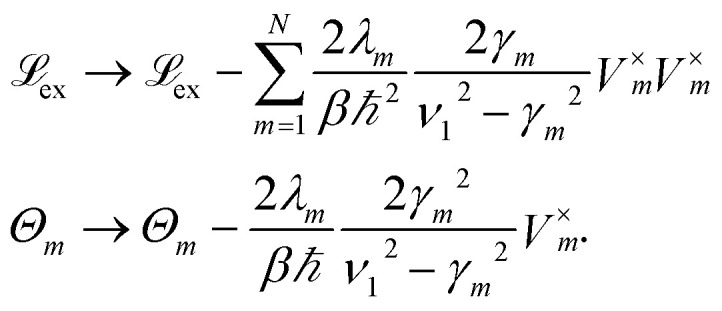
Here *ν*_1_ is defined as the first Matsubara frequency *ν*_1_ = 2π/*βħ*.

For structured spectral densities, a similar hierarchical expansion has been derived which relies on a decomposition of the spectral density in terms of shifted Drude–Lorentz peaks^50,51^ or underdamped Brownian oscillators.^35^ The accuracy of the improved high temperature correction can be validated by various means. For example, convergence could be tested by including more Matsubara frequencies. However, the slow convergence of the Matsubara expansion renders this approach numerically challenging. Less numerically involved approaches include the validation of the stationary state, which is expected to follow a thermal Boltzmann distribution for the parameter regime of C_2_S_2_M_2_. Also, comparison of monomer line-shapes computed with HEOM to analytically known results have been proposed as an appropriate test for the HTA.^38^

## 4 Discussion

Together with LHCII complexes, the C_2_S_2_M_2_ supercomplex aggregates as a large photosynthetic network in the grana membrane. For each C_2_S_2_M_2_ supercomplex, there are about six additional loosely bound LHCII trimers,^4^ which form a large antenna system with densely packed chlorophylls. Energy is either absorbed in the pool of loosely bound LHCII trimers and then transferred to one of the peripherical LHCIIs of the C_2_S_2_M_2_ supercomplex or absorbed directly in the LHCII trimers of C_2_S_2_M_2_. Further, to some extent, energy is absorbed in the minor complexes and PS-II. We expect that the contribution of light absorption in the minor complexes and PS-II to the photosynthetic yield is of less importance, since most of the photoactive area in the grana membrane is covered by the LHCIIs. Thus, to reach a high photosynthetic yield, fast and efficient transfer from the LHCIIs toward the RC core pigments of PS-II becomes indispensable.

In the following, we investigate average transfer times and the efficiency of energy transfer from the peripherical LHCII-m monomeric unit, labeled as unit-1 in Fig. 1, to the reaction center in which the primary step of charge separation takes place. Since the simulation of the complete C_2_S_2_M_2_ supercomplex with HEOM is beyond the current capabilities of *QMaster*, we employ a certain amount of symmetry along the *x*-axis and *y*-axis and reduce the system to a multi-protein network composed of LHCII-m, CP24, CP29, CP47 and the RC-core, comprising 93 pigments in total. This reduction does not take into account transfer pathways between neighboring quadrants of the C_2_S_2_M_2_ supercomplex, which could open additional transfer pathways.

### 4.1 Energy gradient drives directionality

First, we monitor the population dynamics in the absence of trapping and energy losses. We highlight how energy spreads among the different protein complexes, which, as we will analyze in detail, is driven by energy gradients in the pigments of the C_2_S_2_M_2_ supercomplex. Further, we explore the influence of the reorganization process on the exciton dynamics.

In the following, we investigate the deficiency of the approximate combined modified Redfield/generalized Förster method by comparing the population dynamics obtained from the combined method with the HEOM results. The combined modified Redfield/generalized Förster approach divides the exciton Hamiltonian eqn (2) into a strongly coupled part ℋ_strong_ (associated with strongly coupled domains) and a weakly coupled part. Hereby, ℋ_strong_ is given by strongly coupled clusters with inter-site couplings *J*_*nm*_ larger than or equal to a certain threshold value. We follow ref. 11 and use a threshold of 15 cm^−1^. The intra-domain dynamics is then modeled by modified Redfield, while the inter-domain transfer is described by generalized Förster theory. We use the same implementation for the combined modified Redfield/generalized Förster approach as in ref. 38. Therein, transfer time-scales of the Chlb/Chla inter-band relaxation in an LHCII monomer are discussed, and it is shown that the results of the combined modified Redfield/generalized Förster approach are in reasonable agreement with the HEOM results. Since the choice of initial conditions of the combined method is restricted to eigenstates of certain domains in ℋ_strong_, we set the highest energy state of the domain which predominantly populates pigment Chlb 606 of the LHCII-m unit-1 as the initial condition. To allow for comparison, we use the same initial condition for the HEOM calculations.

**Fig. 2:**
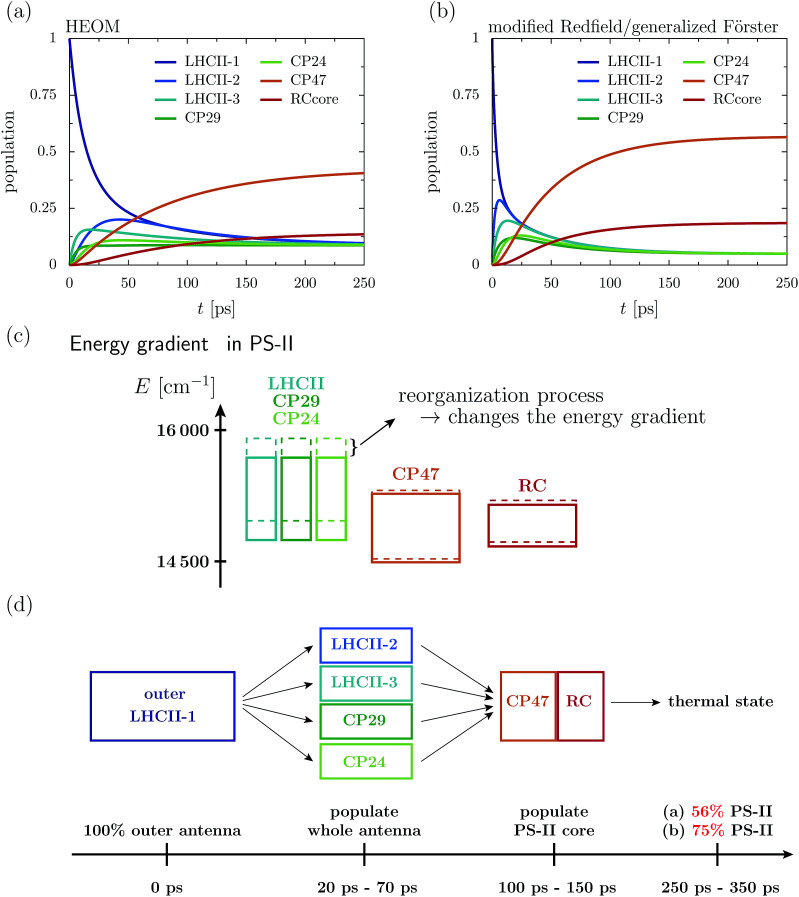
Aggregated populations at *T* = 277 K in absence of trapping in the 93 site network comprising LHCII-m, CP24, CP29, CP47 and the RC-core. The initial state is given by the highest exciton state within the domain of ℋ_strong_, which dominantly excites pigment Chlb 606 of the LHCII-m unit-1. Depicted are the population dynamics within the (a) HEOM and (b) combined modified Redfield/generalized Förster approach. (c) Sketch of the layout of the exciton energy bands before (dashed boxes) and after (solid boxes) dissipation of the reorganization energy. (d) Illustrates rough estimates for the time scales of how energy spreads across the individual proteins.

Fig. 2a depicts the aggregated population at the individual protein complexes obtained within HEOM. Convergence of the hierarchy depth is verified by comparison with a higher truncation level. Further, the stationary state is in good agreement with a thermal Boltzmann distribution, which supports that the high temperature approximation eqn (13) is valid. Details about the convergence of HEOM are given in the ESI.† Overall energy transfer and directionality are driven by energy relaxation along energy gradients within the C_2_S_2_M_2_ supercomplex. LHCII and the minor complexes (modeled by LHCII monomers without Chl 605) exhibit the highest energies, while CP47 and the RC core pigments are lower in energy. The exciton, initially located at the unit-1 LHCII-m monomer, spreads over the complete LHCII-m trimer and populates the minor complexes. The fast initial spread shows as maxima in the aggregated populations at LHCII-m units-2 and 3. The highest population at unit-3 is obtained after about 18 ps, while the maximum population at unit-2 is reached a bit later at about 43 ps. The high population of LHCII-m unit-2 indicates that energy transfer does not exclusively proceed along pathways corresponding to the shortest distance to PS-II and the RC. The minor complexes are populated on a similar time-scale as the monomeric LHCII-m units. On a slower timescale, CP47 and the RC core of PS-II get populated, and finally after about 250–350 ps the system reaches a steady state in which energy relaxation drives the population to the energetically low exciton states at CP47. A schematic sketch summarizing the rough estimates for the energy transfer time-scales is given in Fig. 2d.

The dynamics of the combined modified Redfield/generalized Förster approach (Fig. 2b) diverges from the HEOM results in several aspects. Overall relaxation is overestimated by the combined modified Redfield/generalized Förster approach. In particular, transfer to LHCII-m unit-2 is about seven times faster, and already at 6 ps there is 0.29 population at unit-2. Further, unit-2 gets populated ahead of unit-3. Therefore, the pathway of how energy spreads over the monomeric units of the LHCII-m trimer is reversed when compared to the HEOM calculation, and thus the combined method does not predict reliable pathways of energy flow during the first picoseconds. However, the main difference is in the stationary population, which is not only approached faster (at about 150–250 ps) but predicts a much higher aggregated population at CP47 and the RC. The combined modified Redfield/generalized Förster approach overestimates the efficiency of energy funneling towards the PS-II.

To understand the discrepancy in the stationary population, we need to investigate the energetic layout of the C_2_S_2_M_2_ supercomplex. The stationary state typically follows a thermal Boltzmann distribution. However, the situation becomes more complicated in the presence of the reorganization process, in which the reorganization energy dissipates during the dynamics, which modifies the energetic layout and affects the thermal population. The boxes in Fig. 2c indicate the extension of the exciton bands of the isolated proteins. The dashed lines correspond to the situation where the site energies comprise the bare excitation energy plus the reorganization energy. Due to the reorganization process, the energetic structure changes during the dynamics and the energy of the proteins is lowered by the reorganization energy. In particular, the band of the monomeric LHCII-m units and the band of the minor complexes shift to lower energies, while the small reorganization energies at CP47 and RC induce only minor modifications. In total, the already flat energy gradient gets even more flattened. This has a significant impact on the thermal population. Without the reorganization process (dashed lines), we expect a thermal population of about 0.75 at the pigments of PS-II. Taking into account the reorganization process (solid line) reduces the efficiency of energy funneling and only a population of 0.56 accumulates at PS-II. Our analysis is consistent with the findings for the population dynamics and explains the strong deviations in the stationary state between HEOM and the combined modified Redfield/generalized Förster method. We note that for the combined modified Redfield/generalized Förster method, the effect of the reorganization energy on the thermal population can be corrected by subtracting the reorganization energy from the exciton Hamiltonian prior to the dynamics. This is based on the assumption that the reorganization energy dissipates on an infinitely fast time-scale. With this inclusion of the reorganization process, the combined modified Redfield/generalized Förster approach gives the correct thermal population. Nevertheless, the time-scale of energy relaxation is still overestimated. Further, despite the inclusion of the bath reorganization, the combined modified Redfield/generalized Förster approach does not give an accurate prediction of energy transfer time-scales in the early stages of the transfer process (first ∼ 50 ps). For more details we refer the reader to the ESI.†

### 4.2 The reorganization process affects transfer efficiency

In the following, we investigate how minor changes in the energetic layout of the C_2_S_2_M_2_ supercomplex influence transfer properties such as transfer efficiency and average transfer time. As we have discussed in detail in the previous section, one mechanism is the reorganization process. Here, we continue the discussion and examine how much the reorganization process affects transfer efficiency. Another aspect is the influence of the replacement of the minor complexes with LHCII monomeric units on the transfer properties. For instance, the recently derived exciton Hamiltonian of CP29 shows various differences from the exciton system of an LHCII monomer.^15^

We incorporate the primary step of charge separation by irreversible energy trapping as described in Section 2. Different values for the rate constant of primary charge separation *Γ*_RP1_ have been predicted from fits to fluorescence decay lines, ranging from *Γ*_RP1_^−1^ = 0.1 ps (ref. 12) to *Γ*_RP1_^−1^ = 0.64 ps.^22^ Pump-probe spectra predict even larger time constants for the pheophytin (Pheo) reduction of about 3 ps.^40^ We do not explicitly take into account mechanisms of photoprotection and quenching and phenomenologically describe exciton losses by assuming an exciton lifetime of (*Γ*_loss_)^−1^ = 2 ns.

In the following, we carry out HEOM simulations, in which we include trapping and energy losses. To investigate the effects of the reorganization process on the energy transfer times, we slightly modify the Hamiltonian of the C_2_S_2_M_2_ supercomplex in a benchmark calculation, for which we artificially restore the original energy gradient across the pigment proteins by adding the reorganization energy of 220 cm^−1^ to the site energies of LHCII and the minor complexes. We neglect the minor energetic changes induced by the reorganization process at the pigments of CP47 and the RC and denote the modified Hamiltonian as ℋ_ex,add reorg._. Relaxation time scales in the population dynamics are hardly affected by the shifts in the site-energies, but the thermal state adjusts now according to the modified energy gradient. For ℋ_ex,add reorg._ we obtain a similar thermal state in the population dynamics with high population at the PS-II pigments (0.81), as predicted by the calculations with the combined modified Redfield/generalized Förster method. The small deviations largely result from the fact that we did not add additional reorganization energies to the site energies of CP47 and RC.

**Fig. 3:**
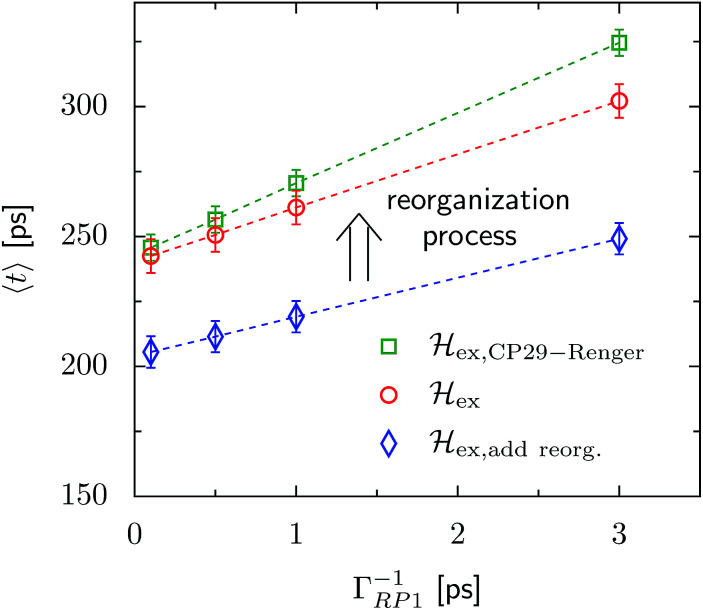
Trapping time evaluated for various rate constants of primary charge separation *Γ*_RP1_ at *T* = 277 K. The transfer time is given as an average over different initial conditions corresponding to eigenstates of the isolated LHCII-m unit-1 monomer. The error bars mark the standard deviations. We investigate changes in the transfer time induced by structural changes in the Hamiltonian. We compare three different scenarios, (i) ℋ_ex,CP29-Renger_, for which we use the CP29 Hamiltonian of Renger *et al.*, ref. 15, (ii) ℋ_ex_, for which the CP29 is substituted by an LHCII monomer (without Chl 605) and (iii) ℋ_ex,add reorg._, for which we add the reorganization energy to the site energies of LHCII-m and the minor complexes of ℋ_ex_.

The transfer time as a function of trapping rate follows a linear trend for the considered parameter regime, as illustrated in Fig. 3. We assume that initially the exciton is located at LHCII-m unit-1, and we populate the initial density matrix according to eigenstates of the isolated LHCII monomeric unit. The shown results correspond to transfer times averaged over all 14 exciton states used as the initial conditions. The chosen initial conditions show similar transfer times, which is also reflected by the small standard deviations (5–6 ps). Transfer times (efficiency) for the C_2_S_2_M_2_ supercomplex (marked by the red circles) are in the range between 242 ps (88.0%) and 302 ps (85.2%), depending on the trapping rate *Γ*_RP1_. ℋ_ex,add reorg._ exhibits more efficient energy funneling towards the pigments at PS-II, and therefore transfer is faster by about 36 ps to 53 ps. Previous calculations based on the combined modified Redfield/generalized Förster method predict transfer times of about 200 ps for transfer from the peripherical domains to the Chl_D1_ in the RC.^22^ This is in good agreement with our results for ℋ_ex,add reorg._, which yields a transfer time of 211 ps for a trapping rate of *Γ*_RP1_ = 0.5 ps, which is similar to the one used in ref. 22.

### 4.3 Minor complex CP29 guides transfer

Since the reorganization process already alters the energy transfer efficiency, we expect that the substitution of the minor complexes by LHCII monomers may also significantly affect the energy transfer properties. In this section, we use the Hamiltonian of CP29 derived by Renger *et al.*, ref. 15, instead of the LHCII monomer replacement. We denote the new Hamiltonian as ℋ_ex,CP29-Renger_, while the previous situation with the LHCII monomer substitution is referred to as ℋ_ex_.

For ℋ_ex,CP29-Renger_, the pigments of CP29 form a lower energy band than the LHCII monomers. This has a two-fold implication for the transfer process. Firstly, the energy gradient between the outer LHCII antenna and the minor complex CP29 gives rise to an additional grade of directionality and supports fast transfer from the peripherical LHCII-m trimer to CP29. The minor complex CP29 presumably acts as an exit marker, which guides energy from the outer antenna towards pigments closer to the reaction center. Secondly, the pigments of CP29 and CP47 form a spatially extended region of low-energy states, and hence energy accumulates at the pigments in proximity to the RC, while the final transfer step to the RC core pigments is energetically uphill and therefore slow. Overall, the two effects result in a slightly slower energy transfer within the C_2_S_2_M_2_ supercomplex while including the CP29 Hamiltonian, see Fig. 3. For large trapping rates, *Γ*_RP1_ > 1 ps, the slow down of the energy transfer gets more pronounced.

**Fig. 4:**
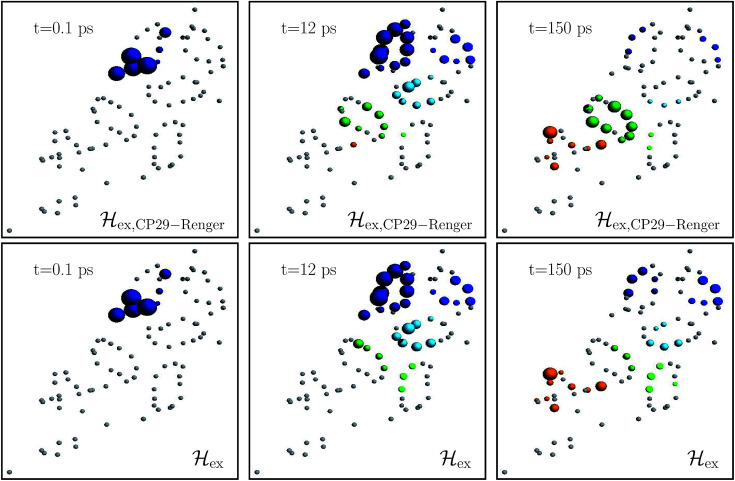
Snapshots of the exciton dynamics in the presence of population trapping in the reaction center (*Γ*_RP1_^−1^ = 0.5 ps) at *T* = 277 K. The spheres represent the positions of the individual pigments while the radii reflect the population at each pigment (we employ an arctan scale). The color signifies the population at the individual protein complexes. Different colors are used for the individual protein complexes (the color encoding is analogues to Fig. 2). Spheres in gray indicate pigments with less than 0.0079 population. As the initial condition, we use the highest eigenstate of the isolated LHCII-m unit-1. The upper and lower panels show the results for the two different Hamiltonians, ℋ_ex,CP29-Renger_ and ℋ_ex_, respectively. Both differ in the structure of the minor complex CP29.

Fig. 4 charts snapshots of the exciton dynamics. The upper (lower) panels correspond to ℋ_ex,CP29-Renger_ (ℋ_ex_). The radius of the colored spheres represents the population at each pigment. For better visualization we use an arctan scale. The spheres are uncolored if the population remains below 0.0079. Initially, the highest eigenstate of LHCII-m unit-1 is excited. Both Hamiltonians show a fast spread of the energy, and at 12 ps the energy distributes across the whole LHCII-m trimer. While ℋ_ex_ distributes population equally among the minor complexes, ℋ_ex,CP29-Renger_ yields a more directed energy transfer towards CP29 and CP47. For longer times of 150 ps, energy accumulates at the low energy states at CP29 and CP47 for ℋ_ex,CP29-Renger_ and thus forms a bottleneck for transfer to the RC. The bottleneck is less pronounced for ℋ_ex_. The RC pigments do not show significant population at any time, since as soon as energy enters the RC there is fast transfer to Chl_D1_ and the fast time-scale of primary charge separation leads to the trapping of the population.

**Fig. 5:**
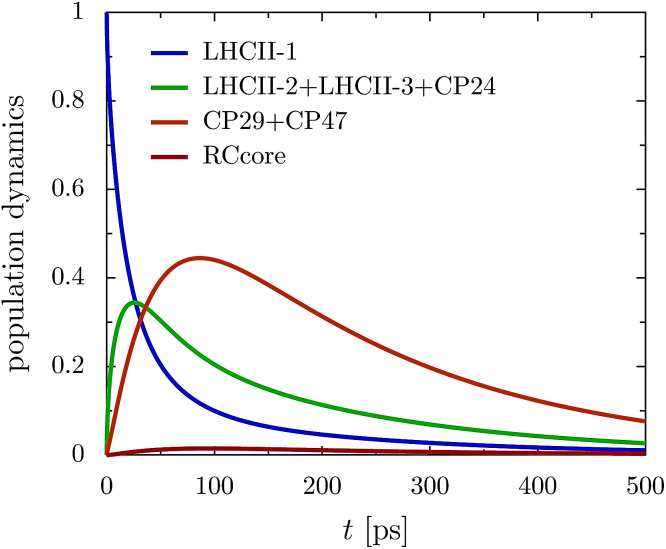
Aggregated populations for ℋ_ex,CP29-Renger_ in the presence of trapping (*Γ*_RP1_ = 0.5 ps) at 277 K. Energy accumulates at low-energy bottleneck states at CP29 and CP47, limiting transfer to the RC.

The rate limiting step in the transfer chain is the energetically up-hill transfer to the RC core. This is illustrated best in the aggregated population dynamics in the presence of trapping in the reaction center, Fig. 5. We obtain a fast decay of population in LHCII-m and after 100 ps, more than 0.75 of the population has left the LHCII-m trimer. At the same time, about 0.44 of the population accumulates in CP29 and CP47. After 300 ps, 0.2 of the population still remains at CP29 and CP47.

## 5 Conclusion

With *QMaster*, a high-performance implementation of the HEOM method, accurate calculations of excitonic energy transfer in multi-protein photosynthetic functional units, such as the C_2_S_2_M_2_ supercomplex, become feasible. We investigate the transfer times and transfer efficiency of energy conversion within the primary step of charge separation.

The general concept behind energy transfer in C_2_S_2_M_2_ is given by energy relaxation. Due to the flat energy gradient across the proteins, even small variations induced by the reorganization of the molecular coordinates within the excited potential energy surface affect the energy transfer process. The impact of the reorganization process is rather significant, and energy relaxation drives much less population to CP47 and the RC than expected from the site energies of the Hamiltonian. The reorganization process induces a noticeable drop in the transfer efficiency of about 1.8% to 2.6% in absolute numbers for a 2 ps exciton lifetime, and thus cannot be neglected in simulations of energy transfer in large multi-protein complexes.

Our simulations suggest that the minor complex CP29 acts as an exit marker and adds directionality to the energy transfer from the peripherical LHCII to the proteins in proximity to the RC core. The C_2_S_2_M_2_ supercomplex is not optimized for efficient transfer. Energy accumulates in low energy states at CP29 and CP47, while the final transfer step needs to overcome an energy barrier and therefore is slow. Thus, the energy transfer exhibits the character of a transfer-to-trap limited model. In conclusion, within our model, we show that the structural layout of C_2_S_2_M_2_ is not optimized for efficient transfer and suggests that photoprotection considerations are very relevant. The extension of accurate HEOM models to this case is possible and is a promising direction for future research. Our accurate simulations provide a first step toward a consistent description of energy transfer in multi-protein networks such as C_2_S_2_M_2_. The currently used parameter sets for the individual proteins are largely obtained empirically by fitting simulations based on approximate methods to experimental spectra, and a refinement of the individual Hamiltonians may be needed. To this end, future works need to focus on the comparison of the accurate HEOM calculations with experimental data, such as absorption spectra, circular dichroism spectra or hole-burning data.^52^

## Supplementary Material

SC-007-C5SC04296H-s001
