# Supplementary material for: Efficiency of energy funneling in the photosystem II supercomplex of higher plants
Source: Chem Sci. 2016 Feb 29;7(7):4174–83. doi: 10.1039/c5sc04296h (PMC6014079; doi:10.1039/c5sc04296h)
Supplement: SC-007-C5SC04296H-s001 [file SC-007-C5SC04296H-s001.pdf]

Electronic Supplementary Material (ESI) for Chemical Science.
This journal is © The Royal Society of Chemistry 2016

1 Parameter of the spectral densities

The coupling of each pigments to the vibrational environment is described by a Drude-Lorentz spectral density

$$J_m(\omega) = 2\lambda_m \frac{\omega\gamma_m}{\omega^2+\gamma_m^2}. \quad (1)$$

The paramter λ_m and γ_m for the individual pigments are listed in Table 1.

Table 1 Parameter for the used spectral densities.

pigment protein	λ	γ^{-1}
LHCII, CP29 and CP24	220 cm^{-1}	15 fs
CP47	38.64 cm^{-1}	50 fs
RC core	50.23 cm^{-1}	50 fs

2 Convergence of HEOM

In order to test convergence of HEOM the dynamics is compared for different truncation levels as is charted in Fig. 1. Initially the highest exciton state of LHCII-m unit 1 monomer is excited. A truncation level of $N_{\max} = 2$ yields sufficient accuracy for the relaxation time-scales.

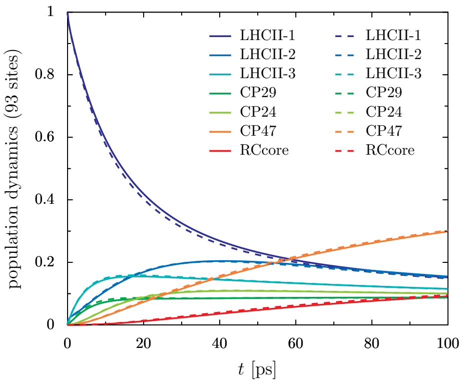

Fig. 1 HEOM results for the aggregated populations at 277 K for different hierarchy truncation levels $N_{\max} = 2$ (solid lines) and $N_{\max} = 3$ (dashed lines).

The high temperature approximation is validated by comparing the stationary state to the expected thermal distribution. The stationary state is extracted at propagation time of 400 ps, after which we consider the aggregated populations as converged. The initial population is set to the highest exciton state of LHCII-m unit 1. The Hamiltonian is given by $\mathscr{H}_{ex}$ (as defined in the main text) and $T = 300$ K. The truncation level of the hierarchy is set to N_{max}=2, for which the hierarchy shows sufficient convergence (see Figure 1). The HEOM calculations show some deviations from the expected thermal

state, but overall we obtain a good agreement with the Boltzmann distribution, which validates the involved high temperature approximation. The results are summarized in Table 2.

Table 2 Comparison of the stationary state (after propagation time of 400 ps) computed with HEOM to the expected thermal aggregated population. The initial condition is set to the highest exciton state in LHCII-1, the Hamiltonian is given by $\mathscr{H}_{ex}$, $T = 277$ K and $N_{max} = 2$.

	thermal population	aggregated population (HEOM)
LHCII-1	0.090	0.088
LHCII-2	0.090	0.088
LHCII-3	0.090	0.088
CP29	0.090	0.087
CP24	0.090	0.087
CP47	0.41	0.42
RC-Core	0.14	0.14

3 Comparison of HEOM with the combined modified Redfield/generalized Förster approach

In this section, we discuss improvements of the implementation of the combined modified Redfield/ generalized Förster approach given in the main text, which is achieved by including the full bath reorganization. The time-scale of the reorganization process is assumed to be infinitely fast, and we subtract the reorganization energy from the site-energies prior to the transfer dynamics. The initial population is given by the highest

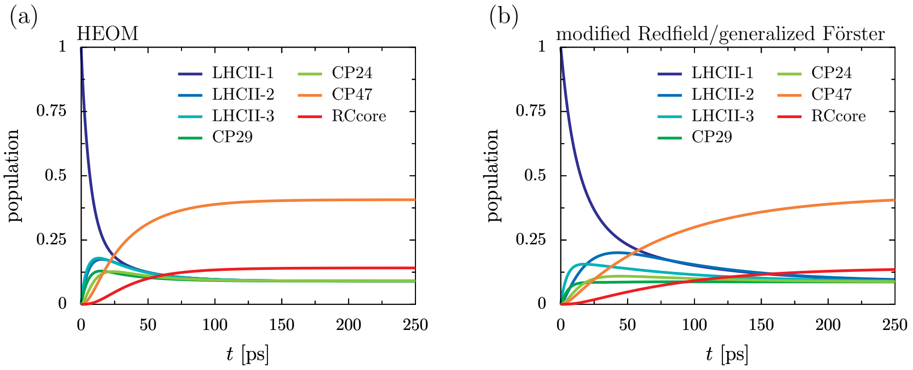

Fig. 2 Aggregated populations at $T = 277$ K in absence of trapping. The initial population is given by the highest exciton state within the domain of $\mathscr{H}_{\text{strong}}$ that populates LHCII-m unit-1. (a) Transfer dynamics obtained with the combined modified Redfield/ generalized Förster approach which includes infinitely fast full bath reorganization by subtracting the reorganization energy from the site-energies prior to the transfer dynamics. (b) shows the corresponding HEOM calculations.

exciton state within the domain of $\mathscr{H}_{\text{strong}}$ that populates dominantly the pigments in the LHCII-m unit 1. The corresponding aggregated populations are shown in Fig. 2. The inclusion of the bath reorganization in the combined modified Redfield/generalized Förster approach ensures that the populations reach the correct thermal state. But, still there are some differences in the dynamics when compared to the HEOM results. The combined modified Redfield/generalized Förster approach overestimates the time-scale of thermal relaxation. Further, the combined modified Redfield/generalized Förster approach does not give an accurate picture of energy transfer in the early stages of the transfer process, and especially until up to the first 50 ps the dynamics differs from HEOM. One deviation, for example, is in the time-scale of how energy spreads within the LHCII-m trimer. The combined modified Redfield/generalized Förster approach predicts almost equally fast energy transfer from LHCII-m unit 1 to unit 2 and unit 3, whereas HEOM predicts a slower energy transfer to unit 2 than to unit 3.
